# Chloroplast genome structure and phylogenetic position of *Syringodium isoetifolium* (Asch.) Dandy

**DOI:** 10.1080/23802359.2021.2003261

**Published:** 2022-02-15

**Authors:** Ying-Gang Ruan, Shuo Yu, Zhang Shun-Hua, Qun-Jian Yin

**Affiliations:** a School of Ecological and Environmental Sciences, East China Normal University, Shanghai, China; b Fourth Institute of Oceanography, Ministry of Natural Resources, Beihai, China; c State Key Laboratory of Estuarine and Coastal Research, East China Normal University, Shanghai, China; d Key Laboratory of Tropical Marine Ecosystem and Bioresource, Fourth Institute of Oceanography, Ministry of Natural Resources, Beihai, China

**Keywords:** *Syringodium isoetifolium*, Illumina sequencing, chloroplast genome, phylogenetic analysis

## Abstract

*Syringodium isoetifolium* (noodle seagrass) is a dioecious perennial seagrass. In this study, the complete chloroplast genome of *S. isoetifolium* was successfully characterized through next-generation sequencing technology. The cp genome was 159,333 bp in length with a GC content of 35.9%, including LSC (89,055 bp), SSC (19,160 bp), and two IRs (25,559 bp). The genome encoded 131 function genes, including 86 protein-coding genes, 37 tRNA genes, and eight rRNA genes. The phylogenetic analysis indicated that *S. isoetifolium* was clustered with *Zostera* and *Ruppia*.

*Syringodium isoetifolium* (Asch.) Dandy is a dioecious perennial seagrass in the Cymodoceaceae. It is distributed in Guangdong, Guangxi, Hainan and Taiwan provinces in China (Zheng et al. 2013). This species often occurs in mixed seagrass beds with other seagrasses species such as *Thalassia hemprichii* (Ehrenb. ex Solms) Asch. (Hydrocharitaceae), *Cymodocea rotundata* Asch. and Schweinf. and *C. serrulata* (R.Br.) Asch. and Magnus (Cymodoceaceae) (Tomasko et al. 1993). Some papers about its screening of microsatellite markers and phylogeography were found (Matsuki et al. 2013; Kurokochi et al. 2016). However, there are no researches about this species chloroplast genome. In this study, we sequenced, assembled and annotated the complete chloroplast genome of *S. isoetifolium* with the next-generation sequencing technology to understand its genomic structure and analyze its phylogenetic position.

Samples of *Syringodium isoetifolium* were collected from Wenchang, Hainan province, China (19.52°E, 110.87°N), and the voucher specimen was deposited in Fourth Institute of Oceanography Herbarium (Shuo Yu, yushuo2005@163.com) under the voucher number WC201906-1. The total genomic DNA was extracted with the modified CTAB method from cleaned shoots (Yu et al. 2020), and sequenced by Illumina Novaseq platform (USA, Illumina company). In total, 4487.1 Mb of raw data and 4389.5 Mb clean data were obtained. The chloroplast genome was assembled with SPAdes v3.9 (Bankevich et al. 2012) and gene annotation was performed via PGA software (Qu et al. 2019).

The complete chloroplast genome length of *Syringodium isoetifolium* was 159,333 bp with a GC content of 35.9%. The cp genome had a typical quadripartite structure including one large single-copy region (LSC) (89,055 bp), one small single-copy region (SSC) (19,160 bp), and a pair of inverted repeats (IRs) (25,559 bp). The genome encoded 131 genes including 86 protein-coding genes, 37 tRNA genes, and eight rRNA genes.

To clarify the phylogenetic position of *S. isoetifolium*, we downloaded 24 completed chloroplast genomes from GenBank database and then aligned them by MATFF v7.308 (Katoh and Standley 2013). The phylogenetic tree was constructed with RAxML software (Stamatakis 2014) using maximum-like method. Bootstrap values were calculated from 1000 replicates analyses. The phylogenetic tree revealed that *S. isoetifolium* was clustered with *Zostera marina* L. (Zosteraceae), *Ruppia sinensis* Shuo Yu and Hartog and *R. brevipedunculata* Shuo Yu and Hartog (Ruppiaceae) (Figure 1). This study was the first time to clearly identify the complete chloroplast of *S. isoetifolium* and can provide useful information for phylogenetic studies of seagrasses.

**Figure 1.**
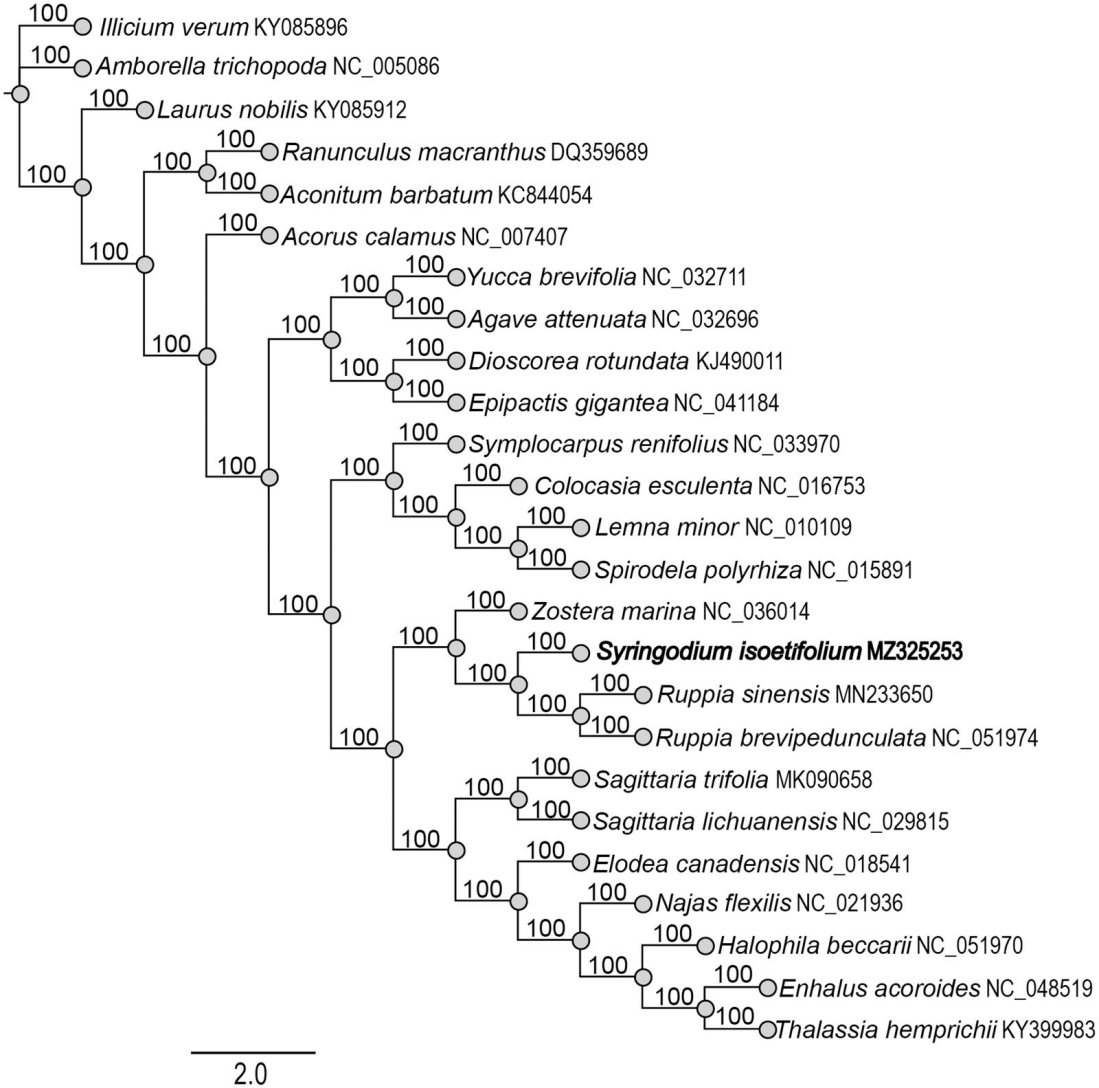
Phylogenetic tree of 25 species based on the chloroplast genome sequences with maximum-likelihood (ML) analysis.

## Data Availability

The chloroplast genome sequence data that support the findings of this study is deposited in the GenBank of NCBI at https://www.ncbi.nlm.nih.gov under the accession number MZ325253. The associated BioProject, SRA, and Bio-Sample numbers are PRJNA755496, SRR15498137, and SAMN20826874, respectively.
